# The Role of Subgenual Resting-State Connectivity Networks in Predicting Prognosis in Major Depressive Disorder

**DOI:** 10.1016/j.bpsgos.2024.100308

**Published:** 2024-03-13

**Authors:** Diede Fennema, Gareth J. Barker, Owen O’Daly, Suqian Duan, Ewan Carr, Kimberley Goldsmith, Allan H. Young, Jorge Moll, Roland Zahn

**Affiliations:** a Centre of Affective Disorders, Institute of Psychiatry, Psychology and Neuroscience, Centre for Affective Disorders, King’s College London, London, United Kingdom; b Department of Neuroimaging, Institute of Psychiatry, Psychology and Neuroscience, King’s College London, London, United Kingdom; c Department of Biostatics and Health Informatics, Institute of Psychiatry, Psychology and Neuroscience, King’s College London, London, United Kingdom; d Cognitive and Behavioural Neuroscience Unit, D’Or Institute for Research and Education, Rio de Janeiro, Brazil; e National Service for Affective Disorders, South London and Maudsley National Health Service Foundation Trust, London, United Kingdom

**Keywords:** Biomarker, Depression, fMRI, Prognosis, Resting-state, Subgenual cortex

## Abstract

**Background:**

A seminal study found higher subgenual frontal cortex resting-state connectivity with 2 left ventral frontal regions and the dorsal midbrain to predict better response to psychotherapy versus medication in individuals with treatment-naïve major depressive disorder (MDD). Here, we examined whether these subgenual networks also play a role in the pathophysiology of clinical outcomes in MDD with early treatment resistance in primary care.

**Methods:**

Forty-five people with current MDD who had not responded to ≥2 serotonergic antidepressants (*n* = 43, meeting predefined functional magnetic resonance imaging minimum quality thresholds) were enrolled and followed over 4 months of standard care. Functional magnetic resonance imaging resting-state connectivity between the preregistered subgenual frontal cortex seed and 3 previously identified left ventromedial, ventrolateral prefrontal/insula, and dorsal midbrain regions was extracted. The clinical outcome was the percentage change on the self-reported 16-item Quick Inventory of Depressive Symptomatology.

**Results:**

We observed a reversal of our preregistered hypothesis in that higher resting-state connectivity between the subgenual cortex and the a priori ventrolateral prefrontal/insula region predicted favorable rather than unfavorable clinical outcomes (*r*_*s*__39_ = −0.43, *p* = .006). This generalized to the sample including participants with suboptimal functional magnetic resonance imaging quality (*r*_*s*__43_ = −0.35, *p* = .02). In contrast, no effects (*r*_*s*__39_ = 0.12, *r*_*s*__39_ = −0.01) were found for connectivity with the other 2 preregistered regions or in a whole-brain analysis (voxel-based familywise error–corrected *p* < .05).

**Conclusions:**

Subgenual connectivity with the ventrolateral prefrontal cortex/insula is relevant for subsequent clinical outcomes in current MDD with early treatment resistance. Its positive association with favorable outcomes could be explained primarily by psychosocial rather than the expected pharmacological changes during the follow-up period.

## Plain Language Summary

Evidence has shown that connectivity of the subgenual cortex, a frontal midline brain region, with 3 other brain regions can predict whether people with never-treated depression benefit more from psychological or medication-based treatments. Here, using resting-state fMRI, we show that subgenual connections with one of these regions, the left ventrolateral prefrontal/insula, also predict future average depression levels in people with difficult-to-treat depression. These data suggest that functional connectivity between these regions may be linked to clinical outcomes in major depressive disorder.

Currently, treatment of major depressive disorder (MDD) is based on a trial-and-error approach, with more than half of patients not responding to their initial antidepressant treatment (1, 2, 3). Multiple treatment gaps at each stage of the care pathway for depression have been identified (4). Identifying prognostic markers of poor clinical outcomes could facilitate stratified treatment algorithms and pathways, but a deeper understanding of the pathophysiology of MDD is needed to develop such prognostic markers.

Functional neuroimaging studies have started to unravel the neural basis of MDD, which is increasingly viewed as a dysfunction of neural networks rather than single brain regions (5, 6, 7, 8, 9, 10). This network perspective is in keeping with leading neuroanatomical models of MDD, which propose that impaired function within prefrontal-limbic neural circuits, particularly the subgenual cingulate cortex and amygdala, explains emotional changes associated with depression (11,12). Neural networks can be observed as fluctuations in blood oxygen level–dependent signal, allowing brain areas related to specific cognitive processes to be identified (13). These fluctuations can be induced by a task or occur spontaneously in the absence of a task, i.e., "resting state," providing insight into the pathophysiology of MDD and potential biomarkers of response to treatment (14,15).

In MDD pathophysiology, resting-state functional connectivity with the subgenual frontal area has consistently emerged as a core component (7, 8, 9, 10,14). Abnormal subgenual frontal region connectivity has been reported in patients with MDD compared with control participants (16, 17, 18) and was associated with symptom severity (19, 20, 21, 22) and duration (23). This is consistent with evidence from positron emission tomography, structural magnetic resonance imaging (MRI), task–based functional MRI (fMRI), and other resting-state fMRI measures (24, 25, 26, 27, 28, 29, 30, 31, 32). Moreover, deep brain stimulation studies targeting the subgenual region have shown promise in treating depression (33), although the results of a recent sham-controlled trial were negative (34). A review (35) showed that the subgenual frontal region was reproducibly associated with individual differences in proneness to self-blaming feelings and is thought to represent affiliative value, particularly in its posterior portion (Brodmann area [BA] 25) and social agency context, particularly in its anterior portion (BA 24/BA 32). It has been proposed that increased functional connectivity of the subgenual frontal region may underpin rumination in MDD, i.e., a tendency to engage in recursive, automatic thoughts often linked to self-critical thinking (36, 37, 38, 39, 40).

Subgenual frontal region resting-state connectivity has also been consistently associated with treatment response (14,41). Interestingly, subgenual frontal region functional connectivity appears to differentially predict response to various forms of treatment. For example, increased baseline connectivity of the subgenual frontal region has been associated with response to transcranial magnetic stimulation (42,43), while reduced baseline connectivity has been associated with response to antidepressant medication (44,45). Notably, Dunlop *et al.* (46) examined whether resting-state subgenual cortex functional connectivity differentially predicted response to cognitive behavioral therapy (CBT) or antidepressant medication at an individual level. In 122 treatment-naïve patients with MDD, higher functional connectivity of the subgenual cortex with the left anterior ventrolateral prefrontal cortex (BA 47)/insula, left ventromedial prefrontal cortex (BA 10), and dorsal midbrain were associated with response to CBT and treatment failure with medication, whereas lower functional connectivity was associated with response to medication and treatment failure with CBT. These findings emphasize the need for more personalized treatment pathways, particularly given the fact that primary care relies mostly on serotonergic medications.

In this preregistered proof-of-concept study (NCT04342299), we sought to determine whether fMRI measures are prospectively associated with clinical outcomes after 4 months in primary care. More specifically, we examined whether the neural signatures of resting-state functional connectivity of the subgenual cortex reported by Dunlop *et al.* (46) generalize to more chronic, treatment-resistant forms of MDD. Because they found that lower connectivity with the subgenual cortex was linked to remission following serotonergic medications and given that UK primary care guidelines recommend changing antidepressant medications in nonresponders (47), we hypothesized that lower functional connectivity between the bilateral subgenual cortex seed as used by Dunlop *et al.* (46) and 1) the left dorsal midbrain, 2) left ventrolateral prefrontal cortex (BA 47)/insula, and 3) left ventromedial frontopolar cortex (BA 10) would be associated with favorable clinical outcomes after 4 months.

## Methods and Materials

### Studies

The design and results of the cluster-randomized ADeSS (Antidepressant Advisor Study) trial (NCT03628027) have been published elsewhere (47,48). This main trial evaluated the feasibility of a novel computerized decision-support algorithm for antidepressant medications in patients with MDD in primary care. Participants who enrolled in the trial were assigned to either 1) use of a computerized decision-support tool by their general practitioner (GP) to assist with antidepressant choices, or 2) treatment-as-usual. Both arms involved standard care because the decision-support tool prompted GPs to follow National Institute for Health and Care Excellence guidelines. However, most participants were recruited outside of the ADeSS main trial through online advertising and received standard care (see Supplement).

As part of an observational substudy within the feasibility trial, participants were invited to attend an optional MRI scan to investigate candidate biomarkers of clinical outcomes after 4 months in primary care. We have published task–based functional imaging results from the same cohort previously (49), but here we report on the resting-state fMRI data for the first time. The study was approved by the National Health Service Health Research Authority and National Research Ethics Service London—Camberwell St Giles Committee (REC reference: 17/LO/2074). All participants provided written informed consent and received compensation for their time and travel expenses. The authors assert that all procedures contributing to this work complied with the ethical standards of the relevant national and institutional committees on human experimentation and with the Helsinki Declaration of 1975, as revised in 2008.

### Participants

As previously described in Fennema *et al.* (49), adults 18 years of age and older were eligible to participate if they fulfilled criteria for an MDD diagnosis according to DSM-5 (50), were currently experiencing a major depressive episode, and had at least moderately severe depressive syndrome on the 9-item Patient Health Questionnaire (score ≥15) (51). Additionally, they had been nonresponders in current or previous episodes to at least 2 serotonergic antidepressants from the following list: citalopram, fluoxetine, sertraline, escitalopram, paroxetine, venlafaxine, or duloxetine. All participants were encouraged to schedule an appointment with their GP to review their treatment and were followed up after 4 months in primary care. Before their medication review, participants completed a resting-state fMRI scan.

Age- and gender–matched control participants without a history of MDD (but including anxiety disorders) and who scored below 10 on the Patient Health Questionnaire-9 depression scale were recruited through online advertising. These control participants completed the same resting-state fMRI scan, which allowed for exploratory cross-sectional comparisons with the MDD group (not preregistered). See the Supplemental Methods for details on inclusion/exclusion criteria, recruitment, clinical assessment, and measures collected.

We considered 3 analytic samples. For the primary imaging analysis, we included 39 participants with current MDD. All met strict criteria for signal dropout, movement, and usable physiological input (see the Supplemental Methods for more details). For the secondary imaging analysis, we also included 4 participants who did not meet the strictest fMRI quality control threshold (reserve list) to assess the impact of lower fMRI quality on the findings, leaving a total of 43 participants. For the tertiary imaging analysis, we compared the MDD group to 16 control participants (15 of whom met the strict criteria and 1 additional control participant who had suboptimal physiological input [reserve list]) (Table S1).

### Primary Outcome

As stated in our preregistered protocol (NCT04342299), we used a continuous measure of clinical outcome rather than categorizing participants into responders and nonresponders using the standard definition of a 50% reduction (52) in Quick Inventory of Depressive Symptomatology—self-rated (16-item; QIDS-SR16) (53) scores due to an unbalanced split between the resulting groups (responders *n* = 9, nonresponders *n* = 34). The outcome was defined as the percentage change from baseline to follow-up on our preregistered primary outcome measure, the QIDS-SR16, where negative percentages corresponded to a reduction in depressive symptoms.

### Image Acquisition

Resting-state echo-planar images were acquired on an MR750 3T MR system (GE Healthcare) with a Nova Medical 32-channel head coil and were optimized for the detection of ventral frontal signal (222 volumes; 41 slices; descending sequential acquisition; repetition time = 2000 ms; echo time = 20 ms; matrix = 64 × 64; field-of-view = 211 mm; flip angle = 75°; slice thickness = 2.9 mm, slice gap = 0.1 mm, interslice distance = 3 mm). Shimming was automatically applied as part of the scanner’s prescan procedures, and 4 additional volumes were acquired and automatically discarded at the start of each fMRI run, allowing for T1 equilibration effects. As demonstrated by measurements of the temporal signal-to-noise, i.e., "the mean of a voxel’s blood-oxygen level–dependent signal over time divided by its standard deviation over time" (54), we obtained good coverage (Figure S1, Table S2).

Participants were shown a fixation cross on the screen and were instructed to keep their eyes open and let their mind wander while focusing on the cross. This setup has been shown to have higher reliability than other eyes-open or eyes-closed instructions (55). Respiration rate and heart rate were monitored. The total scan time was 7 minutes and 24 seconds. For more details on image acquisition, see the Supplemental Methods.

### Regions-of-Interest Selection

A region-of-interest (ROI), seed-based approach was used to assess the resting-state functional connectivity of the subgenual cortex. The a priori subgenual cortex ROI^1^ seed was kindly shared by Dunlop *et al.* (46), who defined it using the Harvard-Oxford Atlas (56) and thresholded it at 50% probability centered on Montreal Neurological Institute (MNI) coordinates ±6, 24, −11. The resulting bilateral subgenual cortex seed comprised two 5-mm radius spheres.

**Figure 1:**
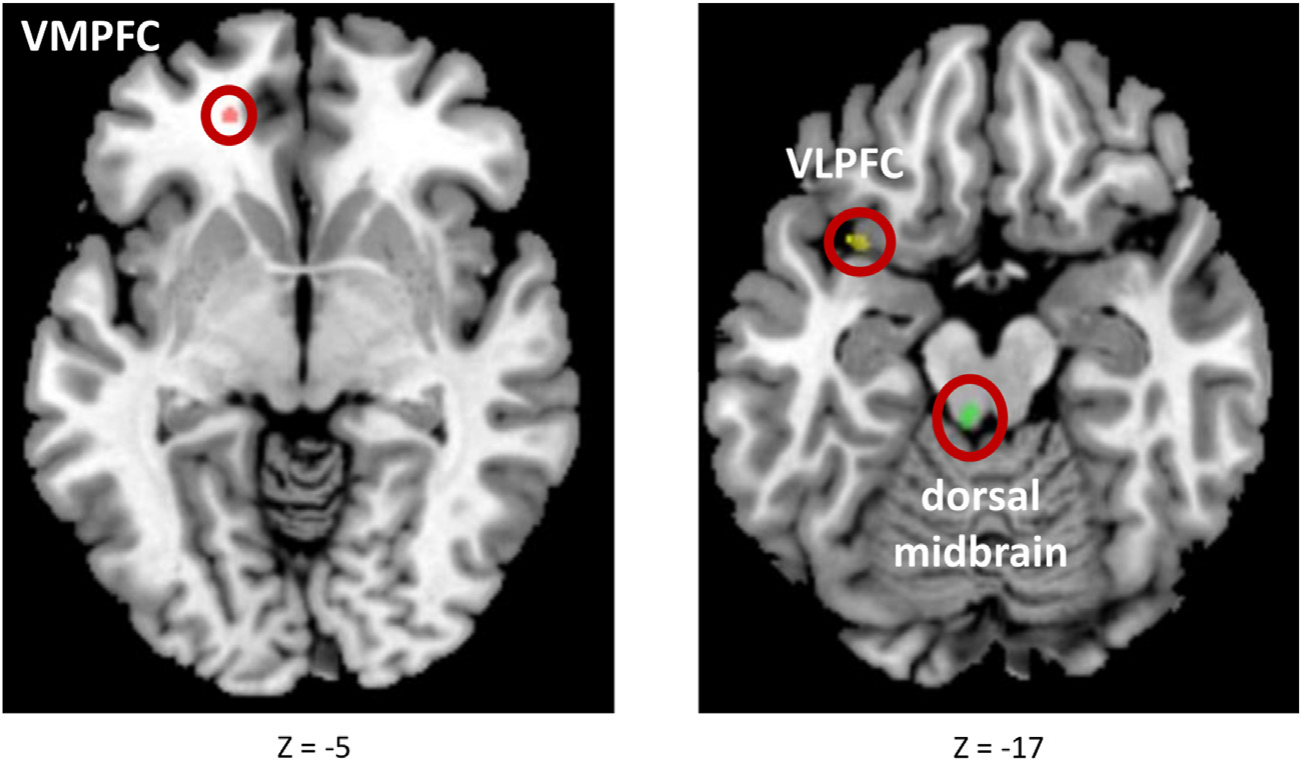
Placement of our 3 preregistered a priori regions-of-interest on the brain. The panels show the preregistered a priori regions-of-interest, as shared by Dunlop *et al.* (46), from which the mean Fisher *z*-transformed correlation coefficients were extracted. Displayed using MRIcron (92). VLPFC, ventrolateral prefrontal cortex; VMPFC, ventromedial prefrontal cortex.

The 3 preregistered a priori ROIs were also kindly shared by Dunlop *et al.* (46), who identified these regions following whole-brain voxelwise subsampling permutation testing to ensure robustness: the left ventromedial prefrontal cortex (BA 10; MNI peak coordinates: −18, 44, −5; 42 1-mm voxel cluster size), left ventrolateral prefrontal cortex (BA 47)/insula (MNI peak coordinates: −31, 12, −17; 77 1-mm voxel cluster size), and dorsal midbrain (MNI peak coordinates: −5, −32, −17; 123 1-mm voxel cluster size) (Figure 1).

### Preregistered Resting-State fMRI Analysis

The resting-state fMRI preprocessing followed a similar approach to that outlined in Workman *et al.* (57), using Data Processing Assistant for Resting-State fMRI Advanced Edition (DPARSF) (58), Artifact Detection Tools (ART) and SPM8. SPM8 was used for preprocessing steps to ensure compatibility with DPARSF.

Functional resting-state echo-planar images and inversion recovery-prepared spoiled gradient echo anatomical images underwent standard preprocessing steps in DPARSF. ART was used to flag spikes in motion, i.e., framewise signal intensity > 3 standard deviations from the global mean and framewise head displacement > 1 mm, and to create nulling regressors. In addition, the MATLAB PhysIO toolbox was used to partially mitigate the impact of physiological noise (59) [version R2021a-v8.0.0, open-source code available as part of the Translational Algorithms for Psychiatry-Advancing Science (60)]. Following this initial preprocessing, echo-planar images underwent linear detrending and nuisance covariates regression [6 motion parameters (61), white matter signal, cerebrospinal fluid signal, ART regressors, and PhysIO regressors] and normalization using nonlinear transformation parameters derived during segmentation. Bandpass filtering was applied to retain frequencies between 0.01 and 0.08 Hz. For more details, see the Supplemental Methods.

Functional connectivity maps were computed using the fully preprocessed functional images for each participant by correlating the average time course within the seed region with the time course of each voxel within the brain, which were Fisher *z*-transformed. Mean Fisher *z-*transformed correlation coefficients over the preregistered a priori cluster-derived ROIs, i.e., the left ventromedial prefrontal cortex and left ventrolateral prefrontal cortex (BA 47)/insula and dorsal midbrain, were extracted for individual participants using the MarsBaR toolbox (62). These were further explored using IBM SPSS Statistics 27.

### Exploratory Resting-State fMRI Analysis

To support the preregistered hypotheses, we compared responders and nonresponders on the extracted mean *z* scores over the preregistered a priori cluster-derived ROIs.

In addition, we entered the Fisher *z*-transformed functional connectivity maps into a 1-sample *t* test in SPM12 to test whether the regression coefficient for QIDS-SR16 percentage change, modeled as a covariate, was different from 0. Voxel-based analyses were thresholded at an uncorrected *p* = .005, and we subsequently used peak voxel-level-based familywise error correction at *p* = .05 over the whole brain and small-volume correction over our a priori defined ROIs.

Lastly, we conducted a 2-sample *t* test to examine differences in subgenual cortex functional connectivity between participants with MDD and controls (see Supplemental Methods).

All analyses were inclusively masked with a gray matter mask based on the default SPM mask.

### Behavioral Data Analysis

All data analyses were carried out using SPSS, with a significance threshold of *p* = .05, 2-tailed. Correlation analysis (Spearman’s rho) was used to investigate the association between the preregistered neural signatures and QIDS-SR16 percentage change, as well as standard clinical variables to investigate their role as potential confounders. The main clinical measures that were collected at baseline and follow-up were the QIDS-SR16 (53), Maudsley Modified Patient Health Questionnaire 9-item scale (63), Generalized Anxiety Disorder 7-item scale (64), Montgomery–Åsberg Depression Rating Scale (65), and Social and Occupational Functioning Assessment Scale (part of the Structured Clinical Interview for DSM-5) (50).

Participants also completed a previously validated computerized task (Moral Sentiment and Action Tendencies) (66, 67, 68, 69), with initial findings reported in Fennema *et al.* (49). Participants were shown written statements describing actions that are counter to social and moral values and were asked to indicate how strongly they would blame themselves and their friend given the unpleasant hypothetical situation, thereby tapping into blame-related emotions. Here, we explored correlations between subgenual resting-state connectivity and blame ratings as a proxy for self-critical thinking. See the Supplemental Methods for more details.

## Results

### Subgroup Characteristics

The MDD and control groups were closely matched on demographic variables (Table S3). There was no evidence for a difference in degree of movement during the resting-state scan or in the content of mind wandering (Table S4). Notably, more than half of the participants engaged in thoughts about others (58%), followed by thinking in spoken words (49%) and thinking about oneself (42%).

Baseline clinical characteristics of the participants with MDD are shown in Table 1 (for control participants, see Table S5). Most participants with MDD fulfilled the DSM-5 anxious distress specifier criteria (81%), often combined with atypical features (47%). Moreover, many participants with MDD met criteria for a lifetime diagnosis of another Axis I disorder (88%), with posttraumatic stress disorder (49%) and other anxiety disorder (40%) being the most common comorbidities.

**Table 1:** Baseline Clinical Characteristics of Participants With MDD (*n* = 43)

	*n* (%) or Mean ± SD; Range
MDD Modified DSM-5 Subtype	
Anxious distress only	9 (21%)
Melancholic features only	0 (0%)
Melancholic features + anxious distress	6 (14%)
Atypical features only	1 (2%)
Atypical features + anxious distress	20 (47%)
No specific subtype	7 (16%)
Age of Depression Onset, Years	17.3 ± 8.7; 4–42
Current MDE Duration, Months	30.2 ± 53.6; 1–231
Number of MDEs	6.8 ± 5.9; 1–30
Illness Duration, Years	26.2 ± 16.7; 2–56
Number of Suicide Attempts	0.5 ± 1.3; 0–6
Maudsley Staging Method	
Mild	18 (42%)
Moderate	25 (58%)
Severe	0 (0%)
Lifetime Axis I Comorbidity	
Posttraumatic stress disorder	21 (49%)
Other anxiety disorder	17 (40%)
Obsessive-compulsive disorder	3 (7%)
Eating disorder	15 (35%)
None	5 (12%)
Family History	
First-degree relative with MDD	15 (35%)
First-degree relative with bipolar disorder	2 (5%)
No family history of MDD	20 (47%)

Percentages may not add up to 100 due to rounding.

MDD, major depressive disorder; MDE, major depressive episode.

As part of the study, participants were encouraged to schedule an appointment with their GP to review their antidepressant medication, which was often a selective serotonin reuptake inhibitor (84%; Table S6). Even though UK care guidelines would recommend changing antidepressant medications in nonresponders, unexpectedly, more than half (53%) did not change their medication, and some even stopped taking their medication (14%; Table S7). On average, participants showed a reduction in both self- and observer-rated depressive symptoms from baseline to follow-up (Table 2 and Figure S2). The percentage change in QIDS-SR16 scores was consistent regardless of medication status (i.e., no change in medication, minimal change, or relevant change; *F*_2,36_ = 0.32, *p* = .73) or any of the other clinical measures at baseline (Table S8). However, there was a positive association between current major depressive episode duration and percentage change in QIDS-SR16 scores (*r*_s__39_ = 0.42, *p* = .01), showing that those with a longer current duration had less favorable clinical outcomes.

**Table 2:** Descriptive Statistics for Clinical Symptom Measures at Baseline and Follow-Up for Participants With MDD (*n* = 43)

	Baseline, Mean ± SD; Range	Follow-Up, Mean ± SD; Range	Difference (95% CI)
QIDS-SR16	17.4 ± 3.4; 10–23	13.6 ± 6.0; 2–24	−3.8 (−5.5 to −2.0)
MM-PHQ-9	18.8 ± 4.4; 8–27	14.3 ± 7.9; 0–27	−4.5 (−6.6 to −2.4)
GAD-7^a^	11.9 ± 4.5; 1–21	10.5 ± 5.9; 0–21	−1.4 (−3.4 to 0.5)
MADRS	31.3 ± 4.9; 22–42	24.2 ± 11.1; 3–44	−7.1 (−10.1 to −4.1)
SOFAS	52.6 ± 6.5; 33–61	56.9 ± 11.8; 33–85	3.9 (1.2 to 6.6)

GAD-7, Generalized Anxiety Disorder, 7 items; MADRS, Montgomery–Åsberg Depression Rating Scale; MDD, major depressive disorder; MM-PHQ-9, Maudsley Modified Personal Health Questionnaire, 9 items; QIDS-SR16, Quick Inventory of Depressive Symptomatology—self-rated, 16 items; SOFAS, Social and Occupational Functioning Assessment Scale.

a Missing follow-up data for 2 participants.

### fMRI Findings

#### Preregistered Analyses

**Figure 2:**
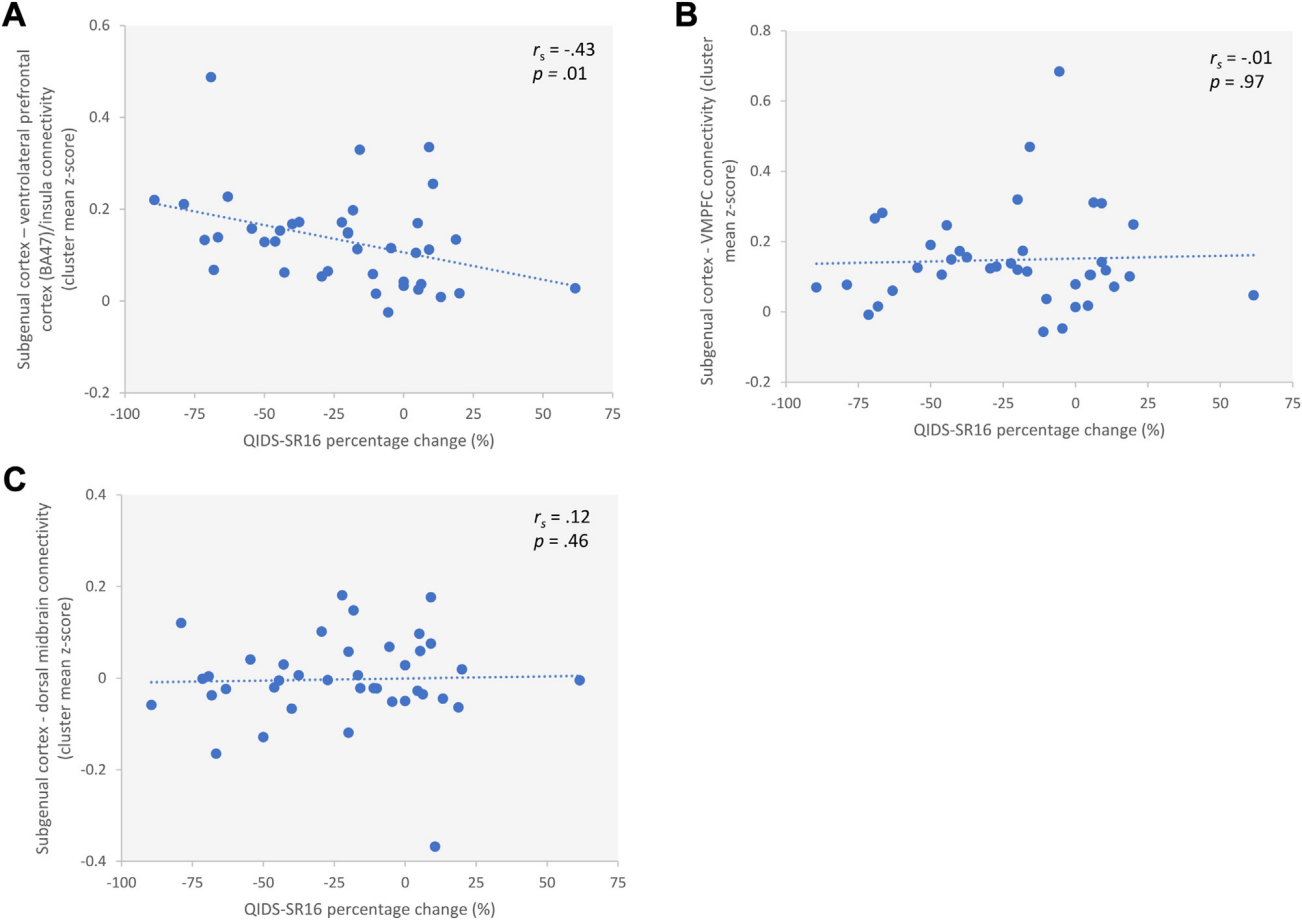
Association between change in depressive score and resting-state connectivity between the subgenual cortex seed region and our preregistered a priori region-of-interest. **(A)** There was a negative association between subgenual cortex–left ventrolateral prefrontal cortex/insula connectivity and Quick Inventory of Depressive Symptomatology—self-rated (16-item; QIDS-SR16) percentage change from baseline to follow-up using the extracted a priori left ventrolateral prefrontal cortex/insula cluster mean *z* scores. **(B)** There was no association between subgenual cortex–left ventromedial prefrontal cortex connectivity and QIDS-SR16 percentage change from baseline to follow-up using the extracted a priori left ventromedial prefrontal cortex cluster mean *z* scores. **(C)** There was no association between subgenual cortex–dorsal midbrain connectivity and QIDS-SR16 percentage change from baseline to follow-up using the extracted a priori dorsal midbrain cluster mean *z* scores. BA, Brodmann area; *r*_*s*_, Spearman correlation; VMPFC, ventromedial prefrontal cortex.

Our preregistered hypotheses were refuted in that lower functional connectivity between the bilateral subgenual cortex seed and our 3 a priori defined ROIs were not associated with favorable clinical outcomes. There was no association between QIDS-SR16 percentage change and the extracted mean *z* scores for the a priori left ventromedial prefrontal cortex ROI (*r*_*s*__39_ = −0.01, *p* = .97) or dorsal midbrain ROI (*r*_*s*__39_ = 0.12, *p* = .46) (Figure 2). However, the ventrolateral prefrontal cortex (BA 47)/insula ROI showed a negative association with QIDS-SR16 percentage change (*r*_*s*__39_ = −0.43, *p* = .006) (Figure 2) that remained when excluding potential outliers (*r*_*s*__38_ = −0.39, *p* = .02) as well as when including the reserve list (*r*_*s*__43_ = −0.35, *p* = .02). In other words, the ventrolateral prefrontal cortex (BA 47)/insula exhibited higher—rather than lower—resting-state connectivity in patients with larger reductions in symptoms after 4 months, i.e., in those with favorable clinical outcomes.

#### Exploratory Analyses

When comparing responders and nonresponders on the imaging measures, there was a significant group difference in subgenual cortex connectivity with the left ventrolateral prefrontal cortex (BA 47)/insula ROI (*t*_37_ = 2.25, *p* = .03) (Figure S3). This was driven by responders showing higher connectivity (mean = 0.19, SD = 0.12) than nonresponders (mean = 0.11, SD = 0.09). There were no group differences in connectivity with the other ROIs, i.e., the ventromedial prefrontal cortex (*t*_37_ = −0.73, *p* = .47) and dorsal midbrain (*t*_37_ = −0.76, *p* = .45) (Figure S3; Supplemental Results).

Whole-brain analyses corrected for small volume within the ROIs revealed a negative association between the a priori ventrolateral prefrontal cortex ROI and QIDS-SR16 percentage change (Figure S4 and Table S9) and no association for the ventromedial prefrontal cortex and dorsal midbrain ROIs (Table S9). Whole-brain voxel-based analysis did not reveal any additional regions associated with clinical outcomes. There was no association between the only potential confounder, current major depressive episode duration, and the neural signature (*r*_*s*__39_ = −0.19, *p* = .24). There were no statistically significant associations between subgenual cortex connectivity and blame ratings, which were used as a proxy for self-critical thinking (Table S10).

The 2-sample SPM model probing group effects (MDD vs. controls) did not show any differences in connectivity with the subgenual cortex seed region at a whole-brain level (Supplemental Results).

## Discussion

We refuted the direction of our preregistered hypothesis that lower resting-state subgenual cortex connectivity would be prospectively associated with favorable clinical outcomes after 4 months in primary care. Contrary to our hypothesis, we found that higher connectivity of the subgenual cortex seed region with the left ventrolateral prefrontal cortex (BA 47)/insula was associated with a greater reduction in depressive symptoms. Interestingly, in Dunlop *et al.* (46), this direction of connectivity was associated with response to CBT—and failure to respond to antidepressant medications. However, more than half of the participants in the current study did not change their treatment or even stopped taking their medication. Therefore, primary care treatment did not resemble active treatment in many cases, and we speculate that response to treatment was likely the result of psychosocial treatment or spontaneous remission, similar to the CBT arm in Dunlop *et al.* (46).

The cluster of activation found in our study was primarily localized in the fronto-insular cortex, which is an area implicated in interoceptive and emotional processing (70,71). Interoception refers to “the representation of the internal world, and includes the processes by which an organism senses, interprets, integrates, and regulates signals from within itself” (70). The fronto-insular cortex is thought to link interoception and emotional feedback, mediating interactions between cortical and limbic brain networks to allow for the integration and regulation of signals from internal and external environments (70,72). Disruption of fronto-insular cortex function has been associated with MDD, potentially reflecting altered interoceptive and self-related processing (73, 74, 75). Moreover, a meta-analysis of fMRI studies identified increased baseline activation of the anterior insula as a predictor of poorer clinical response (15), while positron emission tomography studies have shown that resting-state metabolic activity in the anterior insula differentially predicted response to CBT and antidepressant medication (76,77).

We speculate that resting-state connectivity between the subgenual cortex and fronto-insular cortex may reflect integration of affiliative value, as represented in the posterior subgenual cortex (35), with interoceptive information, as represented in the fronto-insular cortex. Notably, a recent study implicated the fronto-insular region in social information processing, particularly with regard to social domains and appropriateness of behavior (78). It is plausible that connectivity with the subgenual cortex allows for further refinement of affiliative emotional states, which may be important for differentiating the salience of potential threats to affiliative values, for example when violating social norms. Reduced functional connectivity may reflect suboptimal interoceptive and emotional integration, thereby potentially rendering people more vulnerable to overgeneralization of social feelings and rendering patients less responsive to psychotherapies that require conscious reappraisal (79,80). However, we had no behavioral measure of interoception or social domain violations and were therefore unable to directly test this speculative interpretation.

We did not find evidence for an association between clinical outcomes and subgenual cortex connectivity with our other preregistered ROIs, i.e., the ventromedial prefrontal cortex and dorsal midbrain. Furthermore, the voxel-based whole-brain analysis did not show significant effects, which is due to its lower statistical power given the need for multiple comparison correction. The lack of an association of these neural signatures with symptom change could be explained by differences in our study’s design compared with that of Dunlop *et al.* (46), who conducted a randomized controlled trial with treatment-naïve patients who completed a 12-week period of CBT or antidepressant medication. Therefore, functional connectivity between the subgenual cortex and fronto-insular cortex may be more relevant to prognosis in treatment-resistant depression, whereas functional connectivity between the subgenual cortex and ventromedial prefrontal cortex/dorsal midbrain may be more relevant to prognosis in treatment-naïve MDD.

Lastly, we found no evidence of differences in subgenual cortex resting-state connectivity between the MDD group and the control group. This lack of cross-sectional findings might be explained by the small, heterogeneous nature of the current study’s control group, which was recruited as a reference group for the prognostic findings. The control group included participants who met criteria for an anxiety disorder, which has been shown to share similar connectivity disruptions in neural networks as MDD (81). It should also be noted that seed–based resting-state connectivity studies are limited to correlations between a set of predefined ROIs, and it is plausible that using other seed regions or more data-driven methods, such as independent component analysis, would have revealed differences (5). However, a recent seed–based resting-state connectivity study found no difference between patients with remitted MDD who were taking antidepressant medications, medication-free patients with remitted MDD, and matched controls (82).

### Limitations

We did not collect any formal measures of mind wandering or ruminative thinking during the resting-state scan, which could explain the lack of an association between subgenual frontal connectivity and blame ratings as a proxy for self-critical thinking. There is some tentative evidence that individuals with depression engage in more mind wandering than controls, characterized by increased self-focus and self-criticism that can lead to depressive rumination (83,84), and subgenual frontal connectivity has been implicated (37,38). However, we found that patients with MDD and controls did not differ in frequency or content of mind wandering and that participants with MDD were more likely to engage in thoughts about others than thoughts about themselves.

It should be noted that we used a relatively short scan time, which could have limited test–retest reliability and detection of slow frequency dynamics (85). However, the brevity of the scan increases its potential clinical utility and reduces the likelihood of movement while still providing stable estimates of functional connectivity (86). Moreover, our sample consisted mainly of patients with chronic MDD, who often experience anxious distress and other comorbidities. Although these lenient inclusion criteria limit the identification of distinctive features of MDD, we aimed to evaluate the prognostic value of subgenual resting-state connectivity in a pragmatic clinical setting, which is characterized by a high degree of comorbidity between MDD and anxiety disorders (87).

Similarly, treatment in our observational study was not standardized and included a mix of antidepressant medications (selective serotonin reuptake inhibitors, selective norepinephrine reuptake inhibitors, and tricyclics) and psychotherapy. Previous studies have reported specific effects on subgenual frontal region resting-state connectivity following antidepressant medication (45,88) and CBT (89), which means that treatment effects may have introduced variability in the observed neural responses. However, we chose a pragmatic approach by considering primary care as a complex multifaceted intervention, with medication effects as potential sources of prognostic information.

Lastly, we had a relatively modest sample size, which limits generalizability and can result in biased estimates, low reproducibility, and a lack of statistical power to detect small effects (90,91). However, the number of participants was sufficient for a proof-of-concept study to estimate effect sizes (90).

### Conclusions

This study shows the pathophysiological relevance of resting-state subgenual cortex connectivity in current and treatment-resistant MDD. We showed that higher resting-state functional connectivity between the subgenual cortex and fronto-insular cortex is relevant for clinical outcomes. Notably, this neural signature generalized from a treatment-naïve form of MDD to a more chronic, treatment-resistant form of MDD, although unexpectedly in the opposite direction. Further studies are needed to replicate these findings in a controlled trial of CBT versus antidepressant medication in patients with treatment-resistant MDD.

## Supplementary Material

Key Resources Table

## Footnotes

1 Dunlop *et al*. (46) labeled the region subcallosal cingulate cortex.
