# Supplementary material for: The Role of Subgenual Resting-State Connectivity Networks in Predicting Prognosis in Major Depressive Disorder
Source: Biol Psychiatry Glob Open Sci. 2024 Mar 13;4(3):100308. doi: 10.1016/j.bpsgos.2024.100308 (PMC11033067; doi:10.1016/j.bpsgos.2024.100308)
Supplement: Supplement [file mmc2.pdf]

SUPPLEMENTARY INFORMATION

The Role of Subgenual Resting-State Connectivity Networks in Predicting Prognosis in Major Depressive Disorder

Fennema *et al.*

Parts of this Supplementary Online Content have been adapted from a previously published one in Neuroimage:Clinical (doi: 10.1016/j.nicl.2023.103453).

Supplementary Methods

Additional exclusion criteria

General exclusion criteria were: previous prescription of mirtazapine or vortioxetine at therapeutic dose, MRI contraindications, currently receiving specialist psychiatric treatment, high suicide risk on the Mini International Neuropsychiatric Interview (MINI) suicidality screen (1), past diagnosis of schizophrenia or schizo-affective disorder, psychotic symptoms using clinical screening questions, bipolar disorder, at risk of being violent, drug or alcohol abuse over the last six months, suspected neurological condition, pregnancy or insufficient contraception in women of childbearing age and breastfeeding or within six months of giving birth.

Recruitment and clinical assessment

We recruited participants from September 2018 to March 2020 partly through a cluster-randomized feasibility clinical trial, the Antidepressant Advisor Study (ADeSS; NCT03628027). Recruitment was halted due to the COVID-19 pandemic and recommenced in October 2020, using online advertising only, and was completed in August 2021.

As described in the trial protocol (2), general practitioner (GP) practices screened for patients with a history of treatment-resistance to antidepressant medications within their practice, i.e. non-responders to at least two serotonergic antidepressants in the current or previous episodes. Potential participants were approached for consent and, if given, asked to fill in a pre-screening questionnaire. Potentially eligible participants were invited for an in-depth assessment by the study team, which included a clinical assessment using the Structured Clinical Interview for DSM-5 (SCID) to establish a current major depressive disorder (MDD)

(3), a history of participants' depressive episodes, their current and past antidepressant medications, and completing various clinical, behavioral and experimental measures.

A follow-up assessment was conducted to establish whether any changes in baseline measures had occurred. This visit took place around 14-18 weeks after enrolling in the study, which should allow observation of any treatment effect if there is one. The assessment included questions related to medications taken in the study period as well as various clinical and behavioral measures. Please refer to the ADeSS trial protocol for more details regarding these procedures (2, 4).

As the ADeSS trial was stopped due to the COVID-19 pandemic, an alternative recruitment route was employed to continue recruitment for the observational fMRI study. Trial adverts were posted online, with further dissemination of study adverts via university and institutional recruitment circulars. Interested participants were asked to complete a similar pre-screening questionnaire as those approached for the ADeSS trial. If potentially eligible, participants were invited for an in-depth assessment to confirm their eligibility. For more details, please see Fennema (4).

A total of 1,755 participants with a history of MDD showed interest in participating and completed a pre-screening questionnaire. Potentially eligible MDD participants (n = 89) for the ADeSS trial and the fMRI study were invited to attend an in-depth assessment. Of those, 45 participants enrolled in the fMRI study, attended their MRI session and completed the study. Of those 45 participants, ten participants were also part of the ADeSS trial (support tool arm: n = 4; treatment-as-usual arm: n = 6).

Upon study completion, participants in the MDD group were asked to refer partners or friends who might be interested in serving as control participants. Moreover, trial adverts were posted online, with further dissemination of study adverts via university and institutional recruitment circulars. Interested participants were asked to complete a pre-screening

questionnaire targeted to control participants. If potentially eligible, participants were invited for an in-depth assessment to confirm their eligibility and they completed a similar battery of clinical, behavioral and experimental measures as the MDD group.

A total of 350 control participants completed a pre-screening questionnaire, with n = 113 meeting the initial eligibility criteria. Twenty-four control participants were invited for the initial baseline. Following the assessment, n = 22 control participants were enrolled in the study (n = 3 referred by a participant in the MDD group) and n = 20 control participants attended their MRI session.

Imaging criteria

For the primary analysis, all participants met strict criteria for signal dropout (sufficient coverage of the bilateral subgenual cortex, dorsal midbrain, left ventrolateral prefrontal cortex/insula and left ventromedial prefrontal cortex), movement (translation < 3mm; rotation < 2 degrees; less than 10% censored volumes based on framewise signal intensity [> 3 standard deviations from global mean] and framewise displacement [> 1 mm]) and usable physiological input. We chose a threshold of 10% of motion-contaminated volumes as a trade-off between retaining patient data with reasonable quality and avoiding overfitting with too many scanning nulling regressors.

Sample size

A formal power calculation was difficult, with no previous study from which effect sizes could be drawn. As such, this study should be considered as a proof-of-concept for using fMRI to prospectively predict prognosis in MDD. If the neural signatures have at least 70% accuracy, a minimum of n = 44 MDD patients is required to achieve 85% power for a significant prediction of response (p = .05) compared to chance (50%) using a binomial test. Even though a clinically relevant biomarker should show at least 80% accuracy (5), the proposed sample

size is sufficient to determine the feasibility in a subsequent larger sample.

Temporal signal-to-noise ratio

Temporal signal-to-noise ratio (tSNR) was calculated using the following formula [1]:

$$\frac{\bar{S}}{\sigma_N} \quad [1]$$

where $\bar{S}$ is the mean activation signal of the fMRI time series and σ_N the standard deviation of the noise in the time series. Raw values were extracted using the MarsBaR toolbox (6) for our pre-registered *a priori* regions-of-interest (ROI), all of which were kindly shared by Dunlop *et al.* (7):

1. Bilateral subgenual cortex (BA25), as described in Dunlop *et al.* (7). Montreal Neurological Institute (MNI) coordinates: x = +/- 6, y = 24, z = -11; 5mm spheres.
2. Left ventromedial prefrontal cortex (BA10), extracted cluster obtained from Dunlop *et al.* (7).
3. Left ventrolateral prefrontal cortex (BA47)/insula, extracted cluster obtained from Dunlop *et al.* (7).
4. Dorsal midbrain, extracted cluster obtained from Dunlop *et al.* (7).

Image acquisition

High-resolution anatomical images were acquired with a 3D Inversion Recovery prepared Spoiled Gradient Echo (IR-SPGR) sequence (repetition time (TR) = 7.3 ms; echo time (TE) = 3.02 ms; inversion time (TI) = 400 ms; matrix = 256 x 256; excitation flip angle = 11 degrees; field-of-view (FOV) = 270 mm; slice thickness = 1.2 mm, 196 slices). Images for incidental findings review were acquired using a 2D Fast-Recovery Fast Spin-Echo (FRFSE; TR = 4380 ms; TE = 64.85 ms; matrix = 320 x 256; refocusing flip angle = 111 degrees; FOV = 240; 2 mm contiguous slices, 72 slices) and 2D Fluid Attenuated Inversion Recovery (FLAIR)

sequence (TR = 8000 ms; TE = 128.41 ms; matrix = 256 x 128; refocusing flip angle = 111 degrees; FOV = 220; 4 mm continuous slices, 36 slices) and checked for brain abnormalities by a neuroradiologist at King's College London Hospital, independent of additional, internal checks by the study team.

While in the MRI scanner, the participant's head motion was restricted using padding, and heart rate and respiration rate measurements were recorded via a manufacturer-supplied finger pulse sensor (peripheral plethysmograph) and respiratory belt, respectively. A mirror fitted to the head coil allowed participants to view visual stimuli presented during image acquisition, as stimuli were projected onto a screen located behind the participant's head. Verbal instructions were communicated via the MRI intercom, using a pre-defined script to ensure consistency between participants. After the scanning session, participants were asked what they thought about when letting their mind wander.

Image analysis

Functional resting-state echo-planar images (EPIs) and IR-SPGR anatomical images were pre-processed in Data Processing Assistant for Resting-State fMRI Advanced Edition (DPARSF; http://rfmri.org/DPARSFl) (8), i.e. applying slice timing correction, spatial realignment, co-registration of anatomical images to the EPIs, segmentation, normalization (resliced at a voxel size of 3 x 3 x 3 mm) and smoothing, using a kernel of full-width half-maximum equal to 6 mm. Artifact Detection Tools (ART; http://web.mit.edu/swg/software.htm) was used to flag spikes in motion, i.e. framewise signal intensity > 3 standard deviation from the global mean and framewise head displacement > 1 mm, and to create nulling regressors. Participants with spikes in more than 10% of the functional images were deemed to have moved too much and were excluded from the analysis. There is no fixed rule for proportion of spikes above which data should be rejected, but this allowed for a trade-off between retaining patient data with reasonable quality and avoiding overfitting with too many scan-nulling regressors. In addition,

the MATLAB PhysIO toolbox was used to partially mitigate the impact of physiological noise (9) (version R2021a-v8.0.0, open-source code available as part of the Translational Algorithms for Psychiatry-Advancing Science [TAPAS] software collection (10): https://www. translationalneuromodeling.org/tapas). Heart rate and respiration rate measurements were used in a retrospective image correction (RETROICOR) model, using Fourier expansions of different orders for the estimated phases of cardiac pulsation (second order), respiration (second order) and cardio-respiratory interactions (first order) (11). Higher orders of Fourier expansions have been recommended to optimize physiological denoising (12), but this would have resulted in overfitting of the model due to the large number of regressors and limited number of volumes in the current study. The settings used in our study were in line with the original RETROICOR method (11) and represented a trade-off between denoising and overfitting.

Behavioral data analysis

Data were checked for outliers using standardized scores (outside $z = \pm 2.5$ standard deviations from the mean) for the major depressive disorder (MDD) group and the control group separately. Results with outliers were confirmed by supplementary analyses replacing the outlying value by the nearest occurring value in the rest of the sample that was not an outlier. Moreover, data were screened for normal distribution within each group with Kolmogorov-Smirnov tests and if the assumption of normality was violated, non-parametric Mann-Whitney-*U* tests instead of independent sample t-tests were used to investigate between-group differences (MDD vs controls).

Moral sentiment and action tendencies task

In addition to the standard tests, participants completed an experimental, computerized cognitive task, which investigates the neurocognitive underpinnings of blame-related

emotions: the moral sentiment and action tendencies task (MSAT). This task has been validated in previous studies (13-15), but here, we used the modified, shortened version as described in Duan *et al.* (16) and Fennema *et al.* (17).

In short, participants were shown written statements describing actions counter to social and moral values, using Excel Macro or using an online-based version on PsychoPy (18). They were asked to select the emotion that best described how they would feel given the unpleasant hypothetical situation: guilt, shame, contempt/disgust towards self, contempt/disgust towards friend, indignation/anger towards friend, or no feeling/other feeling. Moreover, they were asked to select the action they would most strongly feel like doing: creating distance from self, hiding, apologizing, creating distance from friend, verbally or physically attacking/punishing friend, or no action/other action. Lastly, participants had to indicate how strongly they would blame themselves (i.e. self-blame rating) and how strongly they would blame their friend (i.e. other-blame rating) for the imagined behavior, using a 7-point visual analogue scale, where 1 = not at all and 7 = very much.

MSAT data were checked for completeness, i.e. number of trials in which the participant did not select at least one moral emotion/action tendency. Even though participants were instructed to restrict their choice to only one moral emotion/action tendency, some participants selected more than one choice and such MSAT trials were excluded from the analysis. Participants with less than 80% valid trials were excluded from the overall analysis (n = 4/43).

Supplementary Results

fMRI findings

When categorizing the participants into responders (i.e. participants who showed at least a 50% reduction in depressive symptoms as measured on the Quick Inventory of Depressive

Symptomatology - self-rated (16-items; QIDS-SR16), n = 9) (19) and non-responders (n = 30), responders showed higher subgenual cortex connectivity with the ventrolateral prefrontal cortex/insula (BA47) compared to non-responders (responder: M = .19, SD = .12; non-responder: M = .11, SD = .09; Figure S3). This group difference was significant (t[37] = 2.25, p = .03) and did not change when including the reserve list (t[41] = 2.28, p = .03).

There was no difference in subgenual cortex connectivity between responders and non-responders with the ventromedial prefrontal cortex (t[37] = -.73, p = .47; responder: M = .12, SD = .10; non-responder: M = .15, SD = .15; Figure S3), which did not change when including the reserve list (t[41] = .46, p = .65). There was also on difference for subgenual cortex connectivity between responders and non-responders with the dorsal midbrain (t[37] = -.76, p = .45, partial: M = .00, SD = .07; non-responder: M = .02, SD = .08; Figure S3), which did not change when including the reserve list (t[41] = -.99, = .33).

Exploratory cross-sectional fMRI findings

The two-sample SPM model probing group effects (MDD vs controls) did not show any differences in connectivity with the subgenual cortex seed region, using small-volume correction with our *a priori* ROIs (left ventrolateral prefrontal cortex/insula, left ventromedial prefrontal cortex and dorsal midbrain). These null findings were confirmed for the extracted mean Fisher Z-transformed correlation coefficients for the left ventromedial prefrontal cortex ROI (U[54] = 265.0, z = -.53, p = .60), left ventrolateral prefrontal cortex/insula ROI (t[52] = .31, p = .76) and dorsal midbrain ROI (U[54] = 265.5, z = -.56, p = .57). The findings did not change when including the reserve list, i.e. those participants who did not meet the strictest quality threshold criteria.

Supplementary Tables

Table S1 | Overview of inclusion / exclusion for imaging analysis.

	**MDD**	**Control**	***Total***
Total:	45	20	*65*
Included in main analysis:	39	15	*54*
• Reserve list, applying less stringent movement criteria (translation < 8 mm; rotation < 6 degrees) and suboptimal physiological input	4	1	*5*
Excluded:	2	4	*6*
• Excluded – abnormal images with functional implications	1	1	*2*
• Excluded – excessive movement, but OK coverage	0	2	*2*
• Excluded – excessive dropout, but OK movement	1	1	*2*

MDD = major depressive disorder.

Table S2 | Mean tSNR for regions-of-interest (n=59).

Bilateral subgenual cortex (BA25)	Left ventromedial prefrontal cortex (BA10)	Left ventrolateral prefrontal cortex (BA47)/insula	Dorsal midbrain
137.7	259.5	155.9	105.4

tSNR = temporal signal-to-noise ratio; BA = Brodmann Area.

Table S3 | Baseline demographic characteristics by group.

This table has been adapted from a previously published one in Neuroimage:Clinical (doi: 10.1016/j.nicl.2023.103453)

	**MDD**	**Control**	**Comparison**
	n = 43	n = 16	
**Age**	42.8 ± 15.0; 19 - 73	39.6 ± 12.2; 20 - 66	$t(57) = .76$, $p = .45$
**Gender**			$\chi^2(2,59) = .41$, $p = .81$
Female	n = 35 (81%)	n = 13 (81%)	
Male	n = 7 (16%)	n = 3 (19%)	
Other	n = 1 (2%)	n = 0 (0%)	
**Ethnicity**[a]			$\chi^2(1,58) = 3.54$, $p = .06$
Asian	n = 4 (10%)	n = 0 (0%)	
Black	n = 2 (5%)	n = 0 (0%)	
Other	n = 2 (5%)	n = 0 (0%)	
White	n = 34 (81%)	n = 16 (100%)	
**Native first language**			$\chi^2(1,59) = 1.09$, $p = .30$
English	n = 35 (81%)	n = 11 (69%)	
Non-English	n = 8 (19%)	n = 5 (31%)	
**Years of education**	16.4 ± 3.7; 10 - 24	16.6 ± 3.1; 9 - 22	$t(57) = -.18$, $p = .86$

[a] Missing data for one MDD; categories have been merged into White vs non-White for chi-square test.
Means, standard deviations and range are reported (*M ± SD; minimum – maximum).* Percentages may not add up to 100 due to rounding. * significant at $p < .05$, two-tailed. MDD = major depressive disorder.

Table S4 | Movement parameters and content of mind wandering during resting-state scan by group.

	MDD	Control	Comparison
	n = 43	n = 16	
**Movement parameters**			
RMS translation	.07 ± .05	.07 ± .03	$U(59) = 314.0$, $z = -.51$, $p = .61$
RMS rotation	.07 ± .05	.06 ± .03	$U(59) = 277.0$, $z = -1.14$, $p = .25$
**Mind wandering** [a]			
Number of elements chosen[b]:	2.0 ± 1.2; 0 – 4	1.6 ± 1.5; 0 - 4	$U(59) = 286.0$, $z = -1.01$, $p = .31$
Thinking in spoken words	n = 23 (54%)	n = 6 (38%)	$\chi^2(1,59) = 1.19$, $p = .28$
Thinking in pictures	n = 17 (40%)	n = 6 (38%)	$\chi^2(1,59) = .02$, $p = .89$
Thinking about other people	n = 27 (63%)	n = 7 (44%)	$\chi^2(1,59) = 1.73$, $p = .19$
Thinking about oneself	n = 18 (42%)	n = 7 (44%)	$\chi^2(1,59) = .02$, $p = .90$
None of the above	n = 5 (12%)	n = 2 (13%)	$\chi^2(1,59) = .01$, $p = .93$

[a] Participants were asked to complete a short questionnaire after the fMRI session, to collect ratings on mind wandering during the resting-state scan.

[b] Number of elements chosen, excluding the "none of the above" option.

Means and standard deviations are reported (*M ± SD; maximum - minimum).* Percentages may not add up to 100 due to rounding.
* significant at $p < .05$ threshold, two-tailed. MDD = major depressive disorder; RMS = root mean square.

Table S5 | Baseline clinical characteristics control participants (n=16).
This table has been adapted from a previously published one in Neuroimage:Clinical (doi: 10.1016/j.nicl.2023.103453)

**Past depressive symptoms not meeting MDE criteria**	4 (25%)
**Life-time axis-I disorder using DSM-5 criteria**	
Anxiety disorder	6 (38%)
Subthreshold past posttraumatic stress disorder	2 (13%)
None	9 (56%)
**Family history**	
First degree relative with probable MDD	2 (13%)
No family history of MDD	14 (88%)

Percentages may not add up to 100 due to rounding. MDD = major depressive disorder; MDE = major depressive episode; DSM-5 = Diagnostic and Statistical Manual for Mental Disorders 5th edition.

Table S6 | Current and past MDD treatment (n=43).

This table has been adapted from a previously published one in Neuroimage:Clinical (doi: 10.1016/j.nicl.2023.103453)

**Treatment at baseline**	
**SSRI**	36 (84%)
Sertraline	13 (30%)
Citalopram	10 (23%)
Escitalopram	3 (7%)
Fluoxetine	5 (12%)
Venlafaxine (≤ 150mg)	5 (12%)
**SNRI**	4 (9%)
Duloxetine	2 (5%)
Venlafaxine (> 150mg)	2 (5%)
**Tricyclic antidepressant**	2 (5%)
**Other antidepressant**	1 (2%)
**Add-on treatment**	4 (9%)
**Non-pharmacological treatment**	11 (26%)
**Past treatment**	
1 – 2 medications	30 (70%)
3 – 4 medications	9 (21%)
5 – 6 medications	4 (9%)
**SSRI**	
Sertraline	12 (28%)
Citalopram	21 (49%)
Escitalopram	5 (12%)
Fluoxetine	24 (56%)
Paroxetine	6 (14%)
Venlafaxine (≤ 150mg)	4 (9%)
**SNRI**	
Duloxetine	2 (5%)
Venlafaxine (> 150mg)	1 (2%)
**Tricyclic antidepressant**	4 (9%)
**Other antidepressant**	8 (19%)
**Add-on treatment**	6 (14%)
**Lifetime mental health/psychotherapy service use**	40 (93%)
Of which past secondary care use	10 (23%)

Percentages may not add up to 100 due to rounding. MDD = major depressive disorder; SSRI = selective serotonin reuptake inhibitor; SNRI = selective norepinephrine reuptake inhibitor.

Table S7 | MDD treatment during follow-up period (n=43).
This table has been adapted from a previously published one in Neuroimage:Clinical (doi: 10.1016/j.nicl.2023.103453)

**Main change**	
No change in antidepressant	23 (53%)
Stopped antidepressant	6 (14%)
Lowered dose of antidepressant	0 (0%)
Increase from effective dose to higher effective dose	7 (16%)
Increase from ineffective dose to effective dose	0 (0%)
Change to another antidepressant at effective dose	5 (12%)
Change to another antidepressant at ineffective dose	2 (5%)
**Main antidepressant**	
**SSRI**	30 (70%)
Sertraline	11 (26%)
Citalopram	6 (14%)
Escitalopram	4 (9%)
Fluoxetine	3 (7%)
Venlafaxine (≤ 150mg)	6 (14%)
**SNRI**	4 (9%)
Duloxetine	2 (5%)
Venlafaxine (> 150mg)	2 (5%)
**Mirtazapine**	3 (7%)
**Tricyclic antidepressant**	1 (2%)
**Other antidepressant**	0 (0%)
**Add-on treatment**	5 (12%)
**Change in mental health service use**	
Started accessing mental health service	8 (19%)
Continued care in mental health service	8 (19%)
Stopped mental health treatment	2 (5%)
**Type of mental health service use**	
CBT	3 (7%)
Psychotherapy	5 (12%)
Psychoanalysis	1 (2%)
Counselling	2 (5%)
Other	5 (12%)
**GP appointments related to mental health[a]**	
None	10 (24%)
1	9 (21%)
2	11 (26%)
3	8 (19%)
More than 3	4 (10%)

[a] Missing data for one participant.
Percentages may not add up to 100 due to rounding. MDD = major depressive disorder; SSRI = selective serotonin reuptake inhibitor; SNRI = selective norepinephrine reuptake inhibitor; CBT = cognitive behavioral therapy; GP = general practitioner.

Table S8 | Association between potential clinical confounders and percentage change for primary analysis MDD group (n=39).
This table has been adapted from a previously published one in Neuroimage:Clinical (doi: 10.1016/j.nicl.2023.103453)

		QIDS-SR16 percentage change
**MM-PHQ-9 (baseline)**	rho	.14
	p-value	.41
**GAD-7 (baseline)**	rho	.15
	p-value	.38
**Current MDE duration (months)**	rho	.42*
	p-value	.01
**Age of onset first MDE (years)**	rho	-.29
	p-value	.08
**Number of MDE in lifetime**	rho	-.10
	p-value	.55
**Total duration depression from onset (years)**	rho	.22
	p-value	.18
**Number of suicide attempts**	rho	.08
	p-value	.63

* significant at $p < .05$ threshold, two-tailed. MDD = major depressive disorder; QIDS-SR16 = Quick Inventory of Depressive Symptomatology – self-rated, 16 items; MM-PHQ-9 = Maudsley Modified Patient Health Questionnaire, 9 items; GAD-7 = Generalized Anxiety Disorder, 7 items; MDE = Major Depressive Episode.

Table S9 | Seed-based resting-state subgenual cortex connectivity (n=39).

				MNI peak coordinates				
Hemi-sphere	Region	Cluster size[a]	Brodmann Area	x	y	z	*t* statistic	Voxel-based FWE-corrected *p* value
**Negative association QIDS-SR16 change:**								
left	Fronto-insular cortex	37	47	-30	6	-15	4.50	.001[b]
**Positive association QIDS-SR16 change:**								
NA	No significant regions	NA	NA	NA	NA	NA	NA	NA

[a] In resampled voxels, sized 3x3x3 mm.
[b] Region surviving voxel-based FWE correction over *a priori* ventrolateral prefrontal cortex (BA47)/insula region of interest (peak MNI: x = -31, y = 12, z = -17, Dunlop *et al.* (45)).

QIDS-SR16 = Quick Inventory of Depressive Symptomatology – self-rated, 16 items; FWE = Family-Wise Error; MNI = Montreal Neurological Institute; BA = Brodmann Area.

Table S10 | Association between neural measures and blame-related measures (n=35).

		SC – left VMPFC connectivity	SC – dorsal midbrain connectivity	SC – left VLPFC/insula connectivity
**Self-blame ratings in self-agency**	rho	-.10	-.02	-.08
	p-value	.56	.93	.66
**Other-blame ratings in self-agency**	rho	.12	.06	-.09
	p-value	.48	.74	.62
**Self-blame ratings in other-agency**	rho	-.06	-.12	-.03
	p-value	.73	.50	.86
**Other-blame ratings in other-agency**	rho	.06	.13	-.003
	p-value	.75	.46	.99

* significant at $p < .05$ threshold, two-tailed. Participants were asked to rate how much they would blame themselves (self-blame) or their friend (other-blame) given unpleasant hypothetical situations. They were either the agent (self-agency) or their friend was the agent (other-agency). SC = subgenual cortex; VMPFC = ventromedial prefrontal cortex; VLPFC = ventrolateral prefrontal cortex.

Supplementary Figures

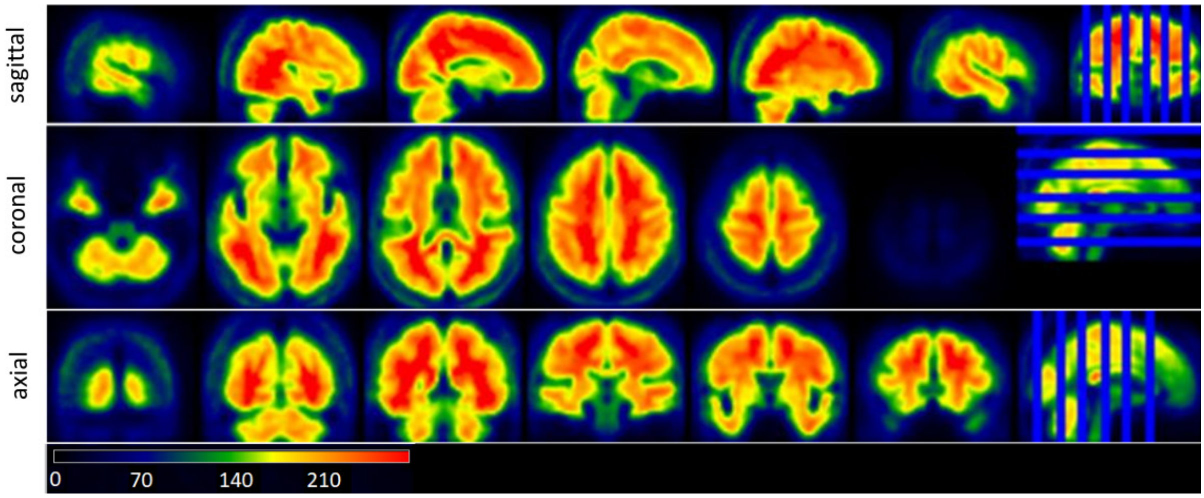

Figure S1 | Overall mean tSNR map across participants for the resting-state scan.

Mean tSNR values for each participant (n = 54) were combined into one overall mean tSNR across participants. The tSNR exceeds the minimum threshold of 40 for most regions as proposed by Murphy et al. (20). Displayed using MRIcron (21). tSNR = temporal signal-to-noise ratio.

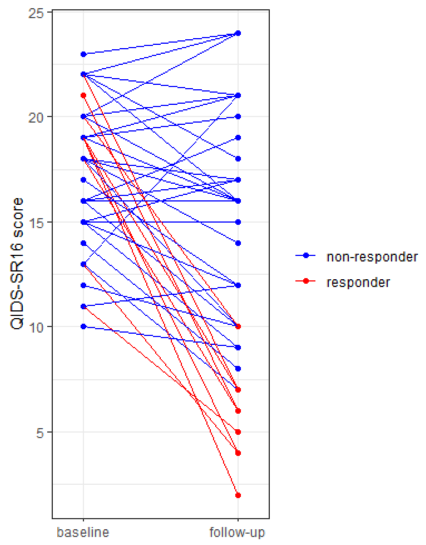

Figure S2 | Change in depressive symptom levels as measured by the QIDS-SR16 (n = 39), grouped by response.

Raw QIDS-SR16 scores at baseline and follow-up (15-18 weeks). Responder was defined as participants who showed at least a 50% reduction in depressive symptoms as measured on the QIDS-SR16. QIDS-SR16 = Quick Inventory of Depressive Symptomatology, self-rated (16-items).

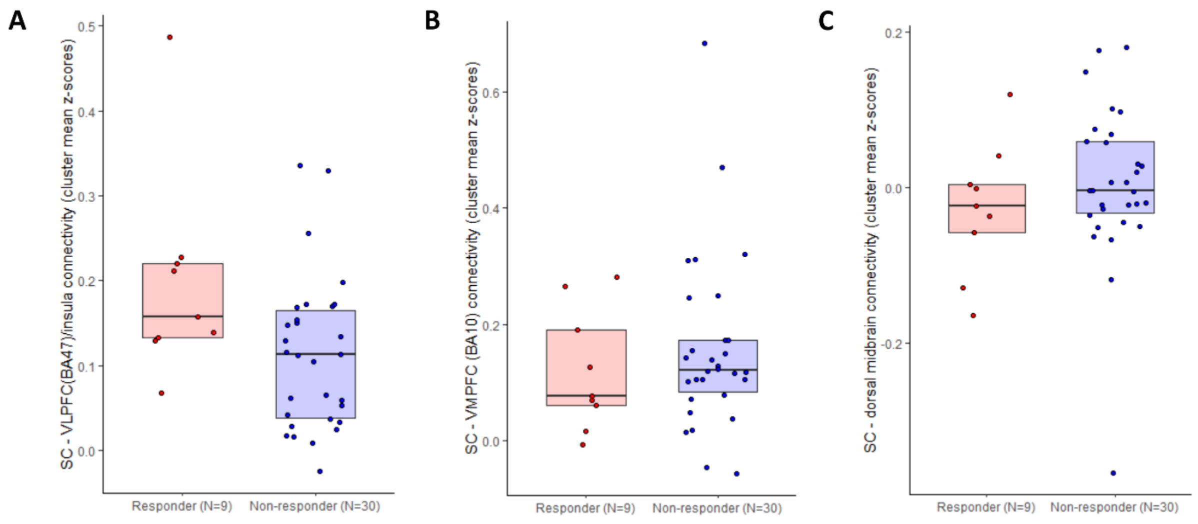

Figure S3 | Comparison between responders (n = 9) and non-responders (n = 30) for resting-state subgenual cortex connectivity with the three *a priori* ROIs.

Panel A) shows that responders, where responder was defined as participants who showed at least a 50% reduction in depressive symptoms as measured on the QIDS-SR16, displayed higher subgenual cortex – left ventrolateral prefrontal cortex (BA47)/insula connectivity relative to non-responders, using the extracted *a priori* left ventrolateral prefrontal cortex (BA47)/insula mean z-scores. **Panel B)** shows that responders and non-responders displayed similar levels of subgenual cortex – ventromedial prefrontal cortex (BA10) connectivity, using the extracted *a priori* left ventromedial prefrontal cortex mean z-scores. **Panel C)** shows that responders and non-responders displayed similar levels of subgenual cortex – dorsal midbrain connectivity, using the extracted *a priori* dorsal midbrain mean z-scores. SC = subgenual cortex; VLPFC = ventrolateral prefrontal cortex; VMPFC = ventromedial prefrontal cortex; ROI = region-of-interest; QIDS-SR16 = Quick Inventory of Depressive Symptomatology, self-rated (16-items); BA = Brodmann Area.

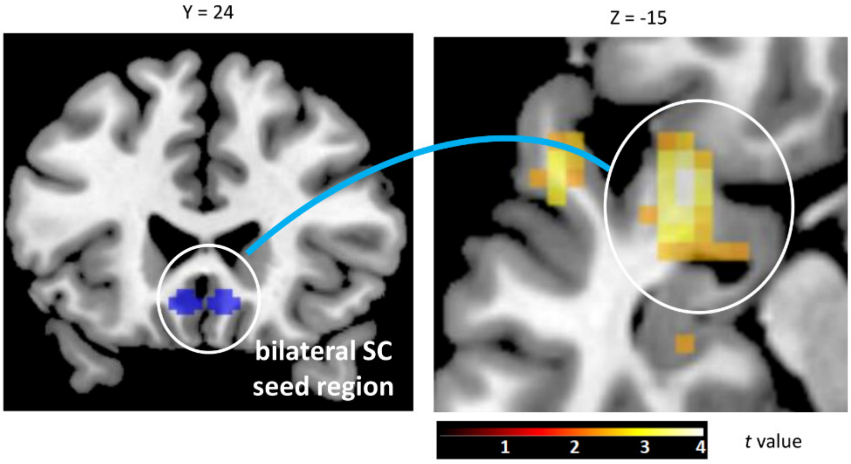

Figure S4 | Voxel-based analysis showing association between change in depressive score and resting-state connectivity between subgenual cortex seed region and left ventrolateral prefrontal cortex/insula.

The figure shows a cropped section through the left ventrolateral prefrontal cortex (BA47)/insula, displayed using MRIcron (21) at an uncorrected voxel-level threshold of p = .005, with no cluster-size threshold (the color bar represents t values; the numbers above the brain slices stand for coordinates of the Montreal Neurological Institute coordinate system). As such, this visualization has not been corrected for multiple comparisons. SC = subgenual cortex; BA = Brodmann Area.

References

1. Sheehan DV, Lecrubier Y, Sheehan KH, Amorim P, Janavs J, Weiller E, et al. The Mini-International Neuropsychiatric Interview (M.I.N.I.): the development and validation of a structured diagnostic psychiatric interview for DSM-IV and ICD-10. J Clin Psychiatry. 1998; 59: 22-33.
2. Harrison P, Carr E, Goldsmith K, Young AH, Ashworth M, Fennema D, et al. Study protocol for the antidepressant advisor (ADeSS): a decision support system for antidepressant treatment for depression in UK primary care: a feasibility study. BMJ Open. 2020; 10(5): e035905.
3. First MB, Williams JBW, Karg RS, Spitzer RL. Structured Clinical Interview for DSM-5 - Research Version (SCID-5 for DSM-5, Research Version; SCID-5-RV, Version 1.0.0). American Psychiatric Association, 2015.
4. Fennema D. Neural signatures of emotional biases and prognosis in treatment-resistant depression. King's College London, 2022.
5. Savitz JB, Rauch SL, Drevets WC. Clinical application of brain imaging for the diagnosis of mood disorders: the current state of play. Mol Psychiatry. 2013; 18(5): 528-39.
6. Brett M, Anton J-L, Valabregue R, Poline J-B. Region of interest analysis using an SPM toolbox. In: 8th International Conference on Functional Mapping of the Human Brain. Neuroimage, 2002.
7. Dunlop BW, Rajendra JK, Craighead WE, Kelley ME, McGrath CL, Choi KS, et al. Functional connectivity of the subcallosal cingulate cortex and differential outcomes to treatment with cognitive-behavioral therapy or antidepressant medication for major depressive disorder. Am J Psychiatry. 2017; 174(6): 533-45.
8. Chao-Gan Y, Yu-Feng Z. DPARSF: A MATLAB Toolbox for "pipeline" data analysis of resting-state fMRI. Front Syst Neurosci. 2010; 4: 13.
9. Kasper L, Bollmann S, Diaconescu AO, Hutton C, Heinzle J, Iglesias S, et al. The PhysIO toolbox for modeling physiological noise in fMRI data. J Neurosci Methods. 2017; 276: 56-72.
10. Frassle S, Aponte EA, Bollmann S, Brodersen KH, Do CT, Harrison OK, et al. TAPAS: An open-source software package for translational neuromodeling and computational psychiatry. Front Psychiatry. 2021; 12: 680811.
11. Glover GH, Li TQ, Ress D. Image-based method for retrospective correction of physiological motion effects in fMRI: RETROICOR. Magn Reson Med. 2000; 44(1): 162-7.
12. Harvey AK, Pattinson KT, Brooks JC, Mayhew SD, Jenkinson M, Wise RG. Brainstem functional magnetic resonance imaging: disentangling signal from physiological noise. J Magn Reson Imaging. 2008; 28(6): 1337-44.
13. Duan S, Lawrence AJ, Valmaggia L, Moll J, Zahn R. Maladaptive blame-related action tendencies are associated with vulnerability to major depressive disorder. J Psychiatr Res. 2022; 145: 70-6.
14. Green S, Moll J, Deakin JF, Hulleman J, Zahn R. Proneness to decreased negative emotions in major depressive disorder when blaming others rather than oneself. Psychopathology. 2013; 46(1): 34-44.
15. Jaeckle T. Neurocognitive basis and treatment of self-blaming emotional biases in major depressive disorder. In: Department of Psychological Medicine. King's College London, 2018.
16. Duan S, Valmaggia L, Fennema D, Moll J, Zahn R. Remote virtual reality assessment elucidates self-blame-related action tendencies in depression. J Psychiatr Res. 2023; 161: 77-83.
17. Fennema D, Barker GJ, O'Daly O, Duan S, Carr E, Goldsmith K, et al. Self-blame-

selective hyper-connectivity between anterior temporal and subgenual cortices predicts prognosis in major depressive disorder. NeuroImage: Clinical. 2023; 39: 103453.
18. Peirce J, Gray JR, Simpson S, MacAskill M, Hochenberger R, Sogo H, et al. PsychoPy2: Experiments in behavior made easy. Behav Res Methods. 2019; 51(1): 195-203.
19. Rush AJ, Trivedi MH, Ibrahim HM, Carmody TJ, Arnow B, Klein DN, et al. The 16-item Quick Inventory of Depressive Symptomatology (QIDS), clinician rating (QIDS-C), and self-report (QIDS-SR): a psychometric evaluation in patients with chronic major depression. Biol Psychiatry. 2003; 54(5): 573-83.
20. Murphy K, Bodurka J, Bandettini PA. How long to scan? The relationship between fMRI temporal signal to noise ratio and necessary scan duration. NeuroImage. 2007; 34(2): 565-74.
21. Rorden C, Brett M. Stereotaxic display of brain lesions. Behav Neurol. 2000; 12(4): 191-200.
